# Healthcare expenditures for people with substance use disorders in drug courts compared to their peers in traditional courts

**DOI:** 10.1016/j.dadr.2024.100258

**Published:** 2024-07-20

**Authors:** Barrett Wallace Montgomery, Arnie Aldridge, Dara Drawbridge, Ira Packer, Gina M. Vincent, Rosa Rodriguez-Monguio

**Affiliations:** a RTI International, Research Triangle Park, North Carolina, RTI International, 3040 Cornwallis Road, Research Triangle Park, NC 27709-2194, United States; b Department of Psychiatry, Center of Excellence for Specialty Courts, University of Massachusetts Medical School, 222 Maple Ave, Chang Building, UMass Chan, Shrewsbury, MA 01545, United States; c Department of Clinical Pharmacy, University of California San Francisco, 521 Parnassus Avenue, San Francisco, CA 94117, United States; d Medication Outcomes Center, University of California San Francisco, School of Pharmacy, 521 Parnassus Avenue, San Francisco, CA 94117, United States; e Philip R. Lee Institute for Health Policy Studies at the University of California San Francisco, 521 Parnassus Avenue, San Francisco, CA 94117, United States

**Keywords:** Substance use disorders, Medicaid, Criminal justice, Healthcare expenditures, Opioid dependence, Two-part model, Drug courts

## Abstract

Individuals within the criminal justice system are at greater risk of substance use–related morbidity and mortality and have substantial healthcare needs. In this quasi-experimental study, we assessed utilization patterns of Massachusetts Medicaid Program (MassHealth) services and associated expenditures among drug court probationers compared to a propensity score–matched sample of traditional court probationers. Risk of reoffending, employment status, age, and living arrangement data were used to calculate propensity scores and match probationers between the two court types, producing a final sample of 271 in each court (N=542). Utilization of services and associated expenditures were analyzed using a two-part model to address the skewed distribution of the data and to control for residual differences after matching from the perspective of the payer (i.e., MassHealth). The largest categories of MassHealth spending were prescription pharmaceuticals, hospital inpatient visits, and physician visits. In the unadjusted analysis, drug court probationers exhibited greater MassHealth services utilization and expenditures than traditional court probationers. However, drug courts enrolled more females, more people at higher risk of reoffending, and more people with opioid use disorders. After controlling for differences between the two court types, the difference in MassHealth services utilization and associated expenditures did not reach statistical significance. Drug court probationers were more likely to engage with healthcare services but did not incur significantly greater expenditures than traditional court probationers after controlling for differences between the samples.

## Highlights

• Pharmaceuticals, hospital care, and physician visits were the largest categories of expenditures in both court types.
• Drug court participation was the best predictor for using any MassHealth services.
• Female sex and older age were the greatest predictors of increased health care expenditures in adjusted analyses.
• Drug court probationers did not incur significantly greater expenditures than their matched peers in traditional court.

## 1 Introduction

Co-occurring drug use and overdose crises have reached new record highs in the United States. While there is a paucity of recent data in many countries worldwide, the United States is experiencing a drug overdose epidemic of unprecedented magnitude compared to the experiences of other high-income countries (Ho, 2019). In 2021, 46.3 million people aged 12 or older (16.5 % of the population) had a substance use disorder (SUD) in the past year, including 6.0 million (2 %) with pain reliever or heroin use disorders (Center for Behavioral Health Statistics and Quality, 2022). A recent analysis estimated the societal costs of the opioid epidemic in the United States at $1.02 trillion in 2017 (Florence et al., 2021). The New England region had some of the highest costs of opioid use disorders per capita (Luo et al., 2021). Likelihood of involvement with the criminal justice system increases as opioid use and misuse increases to levels of dependence, with adjusted odds ratios peaking at 4.2 for heroin use compared to people who do not use opioids (Winkelman et al., 2018).

Traditional approaches such as incarceration have proven ineffective in addressing the treatment needs of individuals with drug-related offenses (Binswanger et al., 2009, Freudenberg and Heller, 2016, Henry, 2020, Massoglia et al., 2014, McNiel et al., 2005). Conversely, drug courts significantly reduce criminal recidivism, incarceration rates, drug use (Rossman et al., 2011, Kearley et al., 2019, Latimer et al., 2006, Marlowe, 2021, Mitchell et al., 2012), and produce positive cost-benefits for taxpayers (Rossman et al., 2011, Marlowe, 2021). Approximately 3.7 million people were on probation in the United States in 2018 (Kaeble and Cowhig, 2018). In the Commonwealth of Massachusetts, 86,000 people were on probation, representing 1.3 % of the state’s population in 2018 (Office of the Commissioner of Probation, 2020).

Drug courts began in 1989 as a response to the increasingly over-burdened criminal justice system in Dade County, Florida, and as a recognition that prison was not serving the rehabilitative needs of the population with SUDs (Belenko, 2001). Since their introduction, adult drug courts have expanded to 1832 courts with an additional 37 dedicated to opioid treatment alone and are present in most counties in the United States (Center, 2023, Marlowe et al., 2016). In the most recent statement from the Office of National Drug Control Policy, the agency has dedicated $95 million to support drug courts for fiscal year 2025 (ONDCP, 2024). Drug courts take a collaborative approach to rehabilitation by combining efforts between probation officers, courts, substance use treatment programs, and other community-based services. Despite the increase of drug courts and research on their effectiveness nationwide, little is known about how drug court probationers healthcare needs, access to and use of services, and associated expenditures differ from in traditional court probationers.

The U.S. government provided health insurance coverage in the form of Medicaid to one in five low-income individuals in 2018 (Kaiser Family Foundation, 2023). Total Medicaid spending in 2018 reached $621 billion with 37.1 % and 37.5 % funded by the federal government and states, respectively (Medicaid and CHIP Payment Access Commission, 2023). One in six dollars in healthcare is spent on Medicaid, and it is the primary source of funding for hospital care, community healthcare, physician offices, and nursing homes. Medicaid may pay a capitation payment per member to private managed care organizations (MCO), or they may pay healthcare providers directly (fee-for-service [FFS]). Medicaid expenditures associated with opioid use disorder in 2013 surpassed $8.4 billion (Leslie et al., 2019).

In 2018, the Massachusetts Medicaid and the Children’s Health Insurance Program (MassHealth) provided health and long-term care coverage to more than 1.8 million low-income residents accounting for one in four Massachusetts residents (MassHealth, 2018). Approximately three out of four MassHealth beneficiaries were enrolled in managed care plans. MassHealth expenditures on healthcare services provision totaled $15.1 billion in 2018 (25 % of total healthcare expenditures), whereas the national average for Medicaid was approximately 17 % (Center for Health Information and Analysis, 2019, Medicaid and CHIP Payment Access Commission, 2023).

In a prior study of this same sample, we examined the rates of recidivism and return to drug use, and utilization and expenditures to the Bureau of Substance Addiction Services (BSAS), the funder of last resort for substance use treatments in Massachusetts. We found no statistically significant differences in rates of recidivism (defined as any new arraignment during the study period, 52 % in drug courts vs 47 % in the traditional courts, *p* >.05) return to drug use (4.3 % positive drug tests for drug court probationers, 3.65 % for traditional court probationers, *p* >.05), or expenditures on treatment services (after controlling for residual differences between the samples, drug court probationers incurred an additional $1447 on average, *p* >.05), however we did find higher utilization of the BSAS system (after controlling for residual differences between the samples, drug court probationers used an additional 36.5 units of service on average) (Rodriguez-Monguio et al., 2021). Because BSAS only captures substance use treatment services among the uninsured, and many states do not have a funding mechanism similar to BSAS, this study aimed to assess total healthcare services utilization and associated expenditures to MassHealth among adults on probation in the Massachusetts Adult Drug Court system compared to a matched cohort of their peers in the traditional court system.

## 2 Methods

### 2.1 Intervention: Massachusetts drug courts

In Massachusetts, each local court system possesses the autonomy to tailor their drug court according to local needs and available resources, but guidance from the state encourages a four-phase structure. The phases include assessment and stabilization, typically lasting between 30 and 90 days; intensive treatment, typically lasting between 6 and 9 months; step-down treatment, typically lasting 6–12 months; and finally, maintenance of recovery, typically lasting 6 months (Executive Office of the Trial Court, 2015).

In the first phase, drug court probationers undergo a full assessment by a local treatment provider and are subjected to multiple and random comprehensive drug and alcohol screenings per week. Drug court probationers will then either be placed in inpatient treatment or meet with their probation officer once a week and attend a weekly or bi-weekly court status hearing before a judge. Drug court probationers receive an assessment of their SUD needs from a clinician to help determine the best course of SUD treatment and an individualized treatment plan is developed for each probationer. These plans consider factors related to the probationer’s clinical needs, prognostic risks, and personal strengths and resources. Both SUD and mental health symptoms are addressed to effectively treat probationers with co-occurring mental health disorders. The treatment plans are comprehensive in addressing both SUD and mental health issues, such as depression, anxiety, and trauma, including post-traumatic stress disorder.

Drug court probationers were considered to have completed the first phase once the treatment provider confirms that the person is in stable recovery, in full compliance with treatment, and self-help programs are in place. In the second phase, drug court probationers continue with the drug and alcohol testing, probation meetings, and court appearances and are typically also living in residential treatment or structured living environments that include a treatment component. Drug court probationers are considered to have completed the second phase after 90 days of negative drug tests, ongoing intensive treatment, and exhibiting pro-social behaviors. In the third phase, drug testing remains as in phases one and two, meetings with the probation officer and court hearings become less frequent, and additional “wrap-around” services (specific to the probationer’s needs) are addressed. After 9 months of negative drug screens, completion of the supervised probation requirements, compliance with treatment, and maintaining employment or school attendance, drug court probationers then apply to proceed to the fourth and final phase. In the final phase, drug court probationers are given more independence to promote self-sufficiency and are subject to less frequent drug tests and less frequent interaction with probation and the court but continue the additional wrap-around services. Requirements to graduate the drug court include completing all four phases; being substance free for at least 12 consecutive months; passing a 90-day hair follicle drug test; approval of the treatment provider; progress toward vocational, educational, and employment goals; a written graduation application; evidence of community service; a suitable residence; a continued care plan; and a sponsor in a 12-step program.

Because each local court system is granted autonomy to tailor their drug court system according to local needs and available resources, and the treatment plans themselves are tailored to each participant, information on the specific services that each participant received is beyond the scope of this study. For more details and an example of one of the court’s treatment plan and requirements, the Massachusetts Adult Drug Court Manual should be consulted (Executive Office of the Trial Court, 2015).

### 2.2 Study design and sample selection

This was an observational quasi-experimental study of the population of drug court probationers (treatment group) in drug courts that were expected to be certified by the state standardization process the year the study commenced (2016) and a matched cohort of traditional court probationers (control group) in the Commonwealth of Massachusetts funded by MassHealth in the period of August 15, 2015, through August 30, 2018. Certification is conducted by the Massachusetts Center of Excellence for Specialty Courts and approved by Chief Justice of the Trial Court (more information on certification can be found in the Massachusetts Adult Drug court Manual; (Executive Office of the Trial Court, 2015)).

The treatment group included six drug courts across the state, certified in 2016 and 2017. The control sample of traditional court probationers was derived from traditional court probation offices that were geographically similar to the six drug courts and did not have a drug court. Later in the evaluation it became apparent that these courts did not have enough high-risk traditional court probationers to match to the drug court group, thus one additional site was added to draw the control group sample. We selected cases for this evaluation based on the date they initiated drug court or traditional probation. This included all new drug court probationers or traditional probationers starting between 8/1/2015 and 2/28/2018 (data collection for baseline cases = 31 months). The traditional court probation control sample was collected from the same date period. Rather than including every traditional court probationer, we selected only those cases with some evidence of a substance use need determined by meeting one of the following criteria: 1.) A clinical SUD diagnosis, 2.) a score of 3 or higher on the Texas Christian University Drug Screen II (TCU Institute for Behavioral Research, 2006), 3) scoring at a moderate to high level on the Ohio Risk Assessment - Community Supervision Tool’s (ORAS-CST) Substance Use Domain Scale (Latessa et al., 2010), 4.) any record of prior substance use treatment, 5.) a high need score on the Risk and Needs Triage (Marlowe et al., 2011), or 6.) reports that an opiate was a primary drug of choice. We followed the sample from court intake to the probation termination date or August 30, 2018, whichever came first. The average follow-up period was 1.3 years (standard deviation [SD]=0.65 years).

We used propensity-score matching to balance the drug court and traditional court probationers to make the groups similar in ways that may be related to recidivism and return to drug use. We attempted to match the groups on the following characteristics, at minimum: age at start of probation, race/ethnicity, gender, level of risk for recidivism (as defined by the ORAS-CST), and evidence of substance use need (as defined above). Some probationers in the full sample were unable to be matched for two reasons: 1. We started with more traditional court probationers, and 2.) Some of these individuals were too low in their risk of reoffending score form the ORAS-CST to be matched to the drug court sample.

### 2.3 Data and measures

MassHealth length of insurance coverage was calculated subtracting each participant’s earliest enrollment date (or the study start date, whichever came first) from the participant’s latest enrollment date (or the end of the study period including follow up, whichever came first). In the study period, probationers were enrolled in the MassHealth program for an average of 23.3 months (SD=9.4 months).

Socio-demographics (sex, age, and homeless status), risk of reoffending (as measured by the ORAS-CST), and prior violent arraignments data were derived from the Massachusetts Trial Court and Criminal Offender Record Information records (Latessa et al., 2010). Trained probation interns abstracted the data independently using a standardized data abstraction form. To ensure data accuracy, we checked study data on an ongoing basis throughout the study.

Data on members, eligibility, enrollment, and medical claims from both MCO and FFS organizations are from MassHealth services. MassHealth data, including services for substance use and mental health disorders and pharmaceuticals, were derived from the Medicaid State Information System. Clinical diagnosis data were derived from the FFS MassHealth dataset and classified using International Classification of Diseases (ICD), Ninth and Tenth Revisions, Clinical Modification codes. Clinical diagnosis analysis included principal, primary diagnosis or any subsequent diagnosis including hospital admitting and discharge diagnosis.

MassHealth treatment services included inpatient, outpatient, home health, residential services, diagnostic services, and prescription medications. Acute care services included inpatient, physician, lab, X-ray, outpatient, clinic, prescription drugs, family planning, dental, vision, and other practitioners’ care. Long-term care services included nursing facilities, intermediate care facilities for the intellectually disabled, mental health, home health services, and personal care support services. MassHealth outpatient mental health services included individual and group therapy, family/couples therapy, and help with a variety of mental health disorders such as trauma, depression, anxiety, eating disorders, and adjustment disorders. Outpatient SUD services included help with withdrawal from substances and support recovery. Prescription drug utilization was assessed using number of days’ supply and refills.

Total healthcare expenditures analysis, including medical and pharmaceutical expenditures (e.g., active ingredient cost and the dispensing fee), included MassHealth-funded services such as Medicare (e.g., premiums paid by MassHealth for Medicare enrollees), Third Party Liability payments (e.g., amount paid by other medical coverage carriers for services covered by MassHealth), and patient payments (e.g., co-payments for outpatient prescription drugs and other medical services). MassHealth payments included the amount allowed for services provided to a beneficiary under a MassHealth benefit plan, such as co-insurance, co-payments, and deductibles, as well as the amount allowed by Medicare for services provided to a beneficiary. All payments were adjusted to 2018 constant prices using the Consumer Price Index for All Urban Consumers (not seasonally adjusted) (U.S. Bureau of Labor Statistics, 2018).

### 2.4 Statistical analysis

Chi-square tests were used to test for significant differences in categorical demographic variables. T-tests were used to test for differences in continuous variables between court types using either pooled or Satterthwaite methods depending on the result of equality of variance tests. Bivariate regression analyses were used to examine potential predictors of MassHealth spending often cited in the literature, such as socio-demographic characteristics, prior violent arraignments, risk of reoffending, and diagnosed opioid use disorders. Statistically significant variables were included in the multivariate regression model while controlling for age at court intake, sex, and healthcare insurance plan to adjust for residual confounding. Analysis of healthcare services utilization and expenditures included all matched probationers, including those who did not use healthcare services. Since we only had measures of utilization for the FFS system services, we used the combined FFS and MCO expenditures in our modeling of whether healthcare expenditures differed significantly by probation system.

We used a two-part regression model due to the highly skewed and zero-inflated data (Diehr et al., 1999). The two-part model first estimated the likelihood of receiving any healthcare treatment service using logistic regression to identify the factors associated with the likelihood of receiving treatment. In the second part, healthcare treatment service expenditures were estimated, conditional on incurring any expenditures, using a generalized linear model with a gamma log link. Average marginal effects and bootstrapped standard errors (BSEs) were calculated using 1000 replications of the model results. We conducted descriptive analyses and bivariate regressions in SAS, Version 15.1 for Windows, and the two-part model in STATA 17 using the TWOPM command (Belotti and Deb, 2015). The Massachusetts Department of Public Health Review Board granted study approval (IRB# 00000701).

## 3 Results

### 3.1 Study population

A total of 639 probationers across court types met study eligibility criteria. The final analytical dataset included 542 probationers—271 in each court type. Out of the 97 unmatched probationers, 43 (44.3 %) received MassHealth services during the study period, whereas 438 (80.8 %) of the probationers included in the matched analyses received MassHealth services (not shown). Most probationers across the court types were white (58.3 %), male (72.3 %), and had prior violent arraignments (73.2 %) (Table 1). One out of eight probationers were homeless at intake (12.5 %) (Table 1). The average age was 32.3 years old (SD=10.1). There were more females in the drug court sample(33.2 %) than in the traditional court sample (12.5 %; *p*<.001) (Table 1), Drug court probationers were significantly younger (mean 30.3 years) than traditional court probationers (mean 34.4 years; *p*<.001), and a significantly greater proportion of drug court probationers (68.4 %) had a clinical diagnosis of an opioid use disorder than traditional court probationers (43.6 %; *p*<.001). There were no significant differences in MassHealth enrollment length.

**Table 1:** Socio-demographics and Clinical Characteristics of Probationers by Court Type, 2015–2018.

	**Traditional Courts**		**Drug Courts**		**Total**		***χ*****2**	**p-value**
	**(#)**	**(%)**	**(#)**	**(%)**	**(#)**	**(%)**		
**Sex**							33.05	<0.001
**Female**	34	12.5 %	90	33.2 %	124	22.9 %		
**Male**	221	81.5 %	171	63.1 %	392	72.3 %		
**Missing**	16	5.9 %	10	3.7 %	26	4.8 %		
**Homeless**							4.71	0.094
**Yes**	41	15.1 %	27	10.0 %	68	12.5 %		
**No**	194	71.6 %	215	79.3 %	409	75.5 %		
**Missing**	36	13.3 %	29	10.7 %	65	12.0 %		
**Prior violent arraignments**							3.99	0.065
**Yes**	208	76.8 %	189	69.7 %	397	73.2 %		
**No**	63	23.2 %	82	30.3 %	145	26.8 %		
**Risk of reoffending at intake**							61.45	<0.001
**Low**	53	19.6 %	37	13.7 %	90	16.6 %		
**Low/Moderate**	9	3.3 %	16	5.9 %	25	4.6 %		
**Moderate**	136	50.2 %	77	28.4 %	213	39.3 %		
**High**	71	26.2 %	100	36.9 %	171	31.5 %		
**Very High**	1	0.4 %	16	5.9 %	17	3.1 %		
**Missing**	1	0.4 %	25	9.2 %	26	4.8 %		
**Opioid use diagnoses**^**¥**^							55.50	<0.001
**Opioid dependence**	60	38.5 %	142	66.0 %	202	54.4 %		
**Opioid abuse**	6	3.8 %	4	1.9 %	10	2.7 %		
**Opioid use**	2	1.3 %	1	0.5 %	3	0.8 %		
**None**	88	56.4 %	68	31.6 %	156	42.0 %		
	**Mean**	**SD**	**Mean**	**SD**	**Mean**	**SD**	**t value**	**p-value**
**MassHealth enrollment months**	23.3	9.4	23.9	9.1	23.6	9.2	−0.71	0.475
**Age at intake**	34.4	11.2	30.3	8.4	32.3	10.1	4.70	<0.001

¥ Based on fee-for-service utility only, total n = 370, 156 in traditional courts and 214 in drug courts,

SD = Standard Deviation

### 3.2 Healthcare expenditures and utilization

In the study period, more drug court probationers (69.7 %) received care through both MCO and FFS plans than traditional court probationers (49.1 %) (not shown). In addition, drug court probationers, 15.9 % received care through MCO only and 9.2 % received care through FFS only, whereas for traditional court probationers, 26.2 % received care through MCO only and 8.5 % received care through FFS only (not shown). Lastly, a greater proportion of traditional court probationers (16.2 %) than drug court probationers (5.2 %) did not use MassHealth services through either MCO or FFS plans (not shown).

**Fig. 1:**
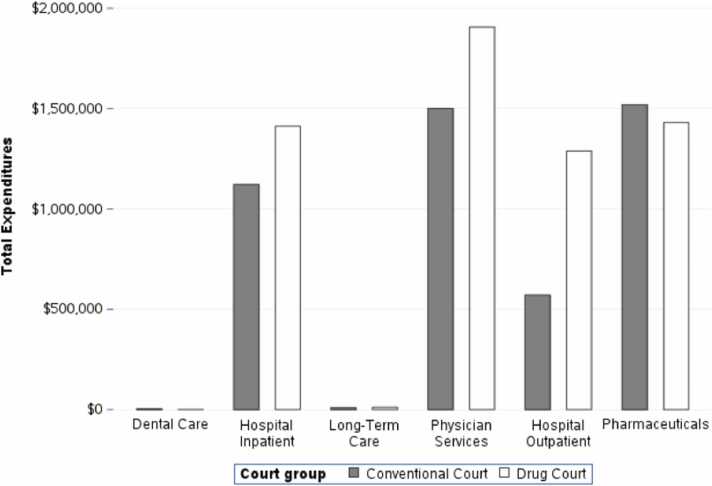
Total Managed Care Expenditures by Service Category and Court Type, 2015–2018.

**Fig. 2:**
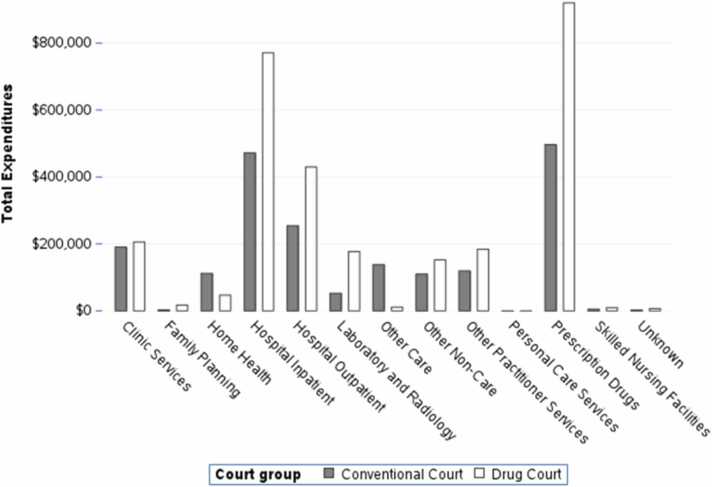
Total Fee for Service Expenditures by Service Category and Court Type, 2015–2018.

After matching, MassHealth expenditures totaled $15,664,774, with expenditures from drug court probationers accounting for $8979,766 and expenditures from traditional court probationers accounting for $6685,008. In MCO plans, prescription drugs, physician visits, and inpatient hospital services accounted for more than 85 % of the MassHealth service expenditures (Fig. 1). In the FFS plans, prescription drugs, inpatient care, and outpatient hospital services accounted for more than 62 % of MassHealth service expenditures (Fig. 2).

Our two-part regression model results are presented in Table 2. Part one of the model revealed significant associations between ORAS-CST risk level and receiving any healthcare treatment services, as well as participation in a drug court. An increase of one level in the ORAS-CST was associated with higher odds of receiving healthcare treatment services (odds ratio=2.42, 95 % confidence interval [95 % CI]: 1.44–4.06; *p*<0.01; Table 2). Moreover, drug court probationers had 11.81 times the odds of using healthcare at least once (95 % CI: 1.41–99.29; *p*<0.05) compared to traditional court probationers (Table 2).

**Table 2:** Likelihood of Receiving Any Healthcare and Adjusted Percent Differences in MassHealth Healthcare Expenditures.

Part one: Likelihood of receiving any healthcare services Log likelihood = −47.92					
	**Odds Ratio**^a^	**Standard Error**	**Z score**	**P>|z|**	**95 % Confidence Interval**
**Drug Court (ref = conventional court)**	**11.81**	**12.83**	**2.27**	**0.02**	**[1.41–99.30]**
Sex (ref = female)	0.77	0.63	−0.32	0.75	[0.15–3.85]
Age (1 unit increase)	1.02	0.03	0.74	0.46	[0.97–1.08]
**Risk level** (1 unit increase)	**2.42**	**0.64**	**3.33**	**0.00**	**[1.44–4.06]**
Intercept	1.35	1.76	0.23	0.82	[0.10–17.33]
Part two: Differences in expenditures conditional on receiving healthcare Log likelihood = −3723.03					
	**Percent difference in costs**^b^	**Standard error**	**Z score**	**P>|z|**	**95 % Confidence Interval**
Drug Court (ref = conventional court)	13.88	15.03	0.98	0.33	[−13.06 – 49.18]
**Sex (ref = female)**	−30.23	**17.35**	−2.26	**0.02**	[−48.83 - −4.88]
**Age (1 unit increase)**	**3.05**	**1.01**	**4.14**	**0.00**	**[1.01–4.08]**
Risk level (1 unit increase)	3.05	7.25	0.46	0.64	[−9.52 – 17.35]
**Intercept**	**54357.19**	**37.71**	**19.75**	**0.00**	**[29194.94–102149.40]**

Note: Significant predictors in bold.

a Odds ratios calculated by exponentiating the beta coefficients from logit part of the model

b Percent difference in costs calculated by first exponentiating the original beta coefficients (log-rate transformation), then subtracting 1 and multiplying result by 100.

Part two of the model revealed that, conditional on using healthcare services, being female was associated with an additional 30 % in expenditures per month on average ($450.66; BSE=153.92; *p*<0.05; Table 2). Furthermore, every additional year of age was associated with a 3 % increase in expenditures per month ($34.44, BSE=9.87; *p*<0.001; Table 2). The average marginal effect of being in the drug court system was $234.78 (BSE=158.36) per month but did not reach statistical significance (*p*=0.14; Table 2).

## 4 Discussion

To our knowledge, this is the first study to assess the MassHealth provision of healthcare services and related expenditures to drug court probationers compared to a matched cohort of traditional court probationers. We found that drug court probationers were more likely to be female, younger, to be moderate to high-risk based on the ORAS-CST, and have a clinical diagnosis of opioid use disorders than traditional court probationers. These findings align with prior research that found that drug courts provide services to a high-risk population in need of substance use treatment (Kearley et al., 2019, Matusow et al., 2013), demonstrating improvements from earlier criticisms that drug courts were screening out people with the greatest needs (Sevigny et al., 2013, DeMatteo et al., 2009).

Of the many cost-benefit analyses conducted over the years, Marlowe summarized the evidence and concluded that the average cost-benefit ratio is between $2 and $4 dollars saved for every $1 invested, however few of these studies consider healthcare costs (Marlowe, 2021). We were only able to find a few studies which evaluated the effects of drug court on healthcare costs. One model-based study estimated a substantial cost of $25,000 per quality-adjusted life year gained when isolating healthcare costs alone (Bernard et al., 2020). Another study was only able to incorporate costs related to mental health service utilization, in which they found the drug court probationers used significantly less mental health services resulting in an overall savings (Logan et al., 2004). Compared to our prior study examining costs to the BSAS system, we found very similar results in the MassHealth system. After adjusting for residual differences between the drug court and traditional court probationers, we found no differences in the adjusted expenditures for substance use treatment services. Yet, similar to these findings on increased likelihood of engagement with the healthcare system, we did find that drug court probationers received nearly 37 more units of service. Also similar to the current study, the differences between the drug court and traditional court probationers, namely risk level, sex, and age, were the significant factors in explaining the differences in expenditures (Rodriguez-Monguio et al., 2021).

Drug court probationers were more likely to have at least one interaction with the healthcare system, suggesting that the drug courts were successful in connecting this population with needed healthcare services. This study found that almost two thirds of MassHealth expenditures funded prescription drug utilization and inpatient and outpatient hospital services for probationers in FFS programs. Likewise, more than four in five dollars were allocated toward funding prescription drugs and physician and inpatient hospital services for probationers in MCO plans, which are appropriate medical services for a population with high levels of SUD. After matching and controlling for any residual differences between individuals in the two court types, the difference in healthcare expenditures was not statistically significant. Drug court probationers used more healthcare services and pharmaceuticals than traditional court probationers without incurring significantly different expenditures after controlling for the differences in the populations.

As states implement expanded Medicaid coverage to address health disparities and opioid treatment programs to cover opioid use disorder treatment services, these study findings may inform policymaking on the healthcare needs and related treatment expenditures for probationers who have SUDs. Our findings could be considered preliminary evidence that, from the perspective of the MassHealth system, drug courts are a net-positive alternative to the traditional court system that increases engagement of drug court probationers with the healthcare system without incurring significant additional medical costs. Our findings also suggest that there is a need to ensure other risk factors, beyond substance use treatment, are also getting addressed in this high-need population. Drug court outcomes may be improved by more effectively tailoring treatment programs to address the specific risk factors identified via the ORAS-CST beyond substance use treatment, such as interventions that focus on challenging criminal thinking and increasing adaptive functioning (Marlowe, 2012). One barrier to this approach of risk-need-responsivity is when judges determine all programming drug court probationers will receive prior to consulting an SUD needs assessment and risk-needs assessment. Use of the risk-needs assessment prior to setting conditions of probation or case plans is a potential policy solution which improves risk-need-responsivity (Drawbridge et al., 2021, Mikolajewski et al., 2021, Vincent et al., 2016).

### 4.1 Limitations

Our study has some limitations. First, drug courts are known to differ significantly from state to state, and within this study, we know that interventions varied between court systems and even within individuals - therefore, these findings may be unique to Massachusetts. Race and ethnicity of the probationers could not be accounted for in the analysis due to a high level of missingness (more than 15 %). While most probationers in this study (59.4 %) received care through both MCO and FFS plans, 21.1 % and 8.9 % of them received care through MCO and FFS plans only, respectively. In addition, 10.7 % did not appear to be enrolled in either MCO or FFS plans. ICD codes and units of utilization were only recorded in the FFS records, limiting our ability to incorporate clinical diagnoses into analyses and make utilization comparisons. Future research should seek to incorporate the role of mental health comorbidities in analyses of drug court outcomes and costs. Type of service was recorded differently between MCO and FFS claims, preventing direct comparison. Since states set up Medicaid payment arrangements for MCO and fees for primary care services, differences in the proportion of probationers who received care through MCO and FFS by court type may contribute to differences in healthcare services utilization and related expenditures. To account for these differences, we used the combined FFS and MCO expenditures in our modeling of whether healthcare expenditures differed significantly by court type. Finally, in March of 2022, the Massachusetts Trial Court reached an agreement with the U.S. Attorney’s Office to resolve allegations that the drug courts violated the Americans with Disabilities Act by discriminating against drug court probationers taking medication for opioid use disorder (U.S. Attorney's Office, 2022). The resulting change in policy, in which only licensed prescribers or opioid treatment programs will make decisions regarding a participant’s treatment plan, could impact the effectiveness of, and healthcare expenditures related to, the drug court system. As this research was conducted prior to the policy change, future research should investigate the possible effects of this change in policy.

By nature of a retrospective study, these study findings do not establish causal effects. Study findings may not be generalizable to other Medicaid programs across the nation or patient populations. In addition, probationers who sought care outside of the Commonwealth were not included in this study. Despite these limitations, this study contributes valuable information for probationers, healthcare providers, policymakers, and researchers on the Medicaid provision of healthcare services and related expenditures in a high-risk and high-need population.

## 5 Conclusions

MassHealth beneficiaries on probation through drug courts in the Commonwealth of Massachusetts had a significantly greater risk of reoffending, were more likely to be female, and had higher rates of opioid use disorders than their peers in traditional courts. Without controlling for these differences in the populations, the drug court probationers did incur greater expenses to MassHealth. However, after controlling for differences between the two groups, these differences are not statistically significant, and therefore not attributable to the drug court system. Drug court probationers were also more likely to have at least one interaction with the healthcare system, a desired outcome. Taken together, study findings suggest that drug court probationers may have increased access to healthcare services, while at the same time not adding significantly to expenditures. Future research should focus on this high-need category of probationers with SUDs and associated mental and behavioral health comorbidities.

## Role of funding source

This research was funded by the Massachusetts Executive Office of the Trial Court contract #2019-28332. The Massachusetts Executive Office of the Trial Court did not participate in any aspects of the research process, drafting or editing this manuscript. The findings, interpretations, and conclusions expressed in this manuscript are entirely those of the authors, and do not represent the views of the Massachusetts Executive Office of the Trial Court. The authors alone are responsible for the content and writing of this manuscript.

## Declaration of Competing Interest

none
